# A Case of Tungiasis in a Greek Patient: A Tropical Souvenir

**DOI:** 10.7759/cureus.77910

**Published:** 2025-01-24

**Authors:** Theodora Douvali, Theodora Zafeiropoulou, Georgios Sarris, Anna Danai Panopoulou, Panorea Georgia Antonatou, Vasiliki Chasapi, Stella Eugenie Chryssou, Dimitrios Motsios, Aristeidis Molympakis, Evangelia Theofano Piperaki

**Affiliations:** 1 Department of Dermatology and Venereology, Andreas Syggros Hospital, Athens, GRC; 2 Department of Microbiology, Andreas Syggros Hospital, Athens, GRC; 3 Department of Microbiology, School of Medicine, National and Kapodistrian University of Athens, Athens, GRC

**Keywords:** cutaneous parasitosis, flea, tanzania, travel history, tunga penetrans, tungiasis

## Abstract

Cutaneous parasitosis caused by fleas belonging to the *Tunga* genus is a common disease in certain regions of the globe like Latin America or Sub-Saharan Africa, but rare in western countries. Its clinical course varies from self-limiting disease to severe systemic infection, making it clear that early diagnosis and treatment are of great importance. We describe a case of imported tungiasis of the lower limb in a patient with a recent travel to Tanzania. The patient presented with dark lesions on the toes, pain, oedema, and pruritus. Clinical and dermoscopic evaluation raised the suspicion of parasitic infection. The lesions were removed surgically, and microscopic and pathologic examination of the tissue revealed the *Tunga penetrans* flea and its eggs, confirming the diagnosis. The procedure was followed by antibiotic treatment and the infection was resolved without complications. Given the rarity of tungiasis in developed countries, a careful clinical inspection in combination with a detailed history including travel history is vital to suspect such diseases. Laboratory and pathological findings are important to establish the diagnosis and lead to early treatment and complication avoidance.

## Introduction

Tungiasis is a parasitic cutaneous disease caused by the female sand flea species, *Tunga penetrans*, and rarely *Tunga trimamillata*, insects that affect hosts by penetrating from the outer cutaneous layers; hence, its classification as ectoparasitosis [1]. Tungiasis is endemic in Latin America, the Caribbean, and Sub-Saharan Africa, and inadequate housing, animal domestication in its vicinity, and poor general and health education have been shown to contribute to the prevalence of the disease [2]. Tungiasis predominantly affects the lower limbs, especially the toes and subungual and periungual areas, and commonly presents in the form of single or multiple papules or nodules [3,4]. It is usually acquired by walking barefoot on the sandy soil of endemic regions [5].

Although it can be self-limited, it may lead to serious debilitating disease, especially in residents of endemic areas. Tungiasis also affects children more heavily, given their intense outdoor activities, along with the elderly, a population with exacerbating vascular and immunological comorbidities that impair healing and immunocompromised hosts with diminished defences against infestation. In some cases, hospitalization or even admission to an intensive care unit is required, due to complications such as gangrene, osteomyelitis, and toe amputation [2,3,6]. Tungiasis is rare in non-endemic areas. It is clearly associated with travel and the duration of stay in the case of international tourists, an ever-growing population [7]. 

The prevalence of tungiasis in travelers is not known as most cases are not formally recorded. In a French survey, 4.2% of patients with skin diseases returning from tropical areas were diagnosed with tungiasis [8]. As the diagnosis is primarily clinical and the clinicians in non-endemic countries are not familiar with the disease, tungiasis is commonly misdiagnosed. This may lead to delayed diagnosis and increased morbidity [4,9]. A large list of diseases is included in the differential diagnosis. Furthermore, the diagnosis becomes more challenging, as many patients present with already manipulated lesions, with secondary bacterial or fungal infections [10,11]. Laboratory examinations and biopsy of the lesion are usually important to establish a diagnosis [12].

Herein, we report a case of tungiasis in a Greek woman with a history of recent travel to Tanzania.

## Case presentation

A 24-year-old Caucasian woman, otherwise healthy, working in the field of occupational therapy, presented with growing brown lesions on the distal phalanges of the right second, third, and fourth toe. No medical history, surgeries, allergies or medication were reported. The patient also complained about swollen toes with accompanying pain, pruritus, and a foreign body sensation. The symptoms began two days after returning from a 20-day trip to Tanzania (Africa). The patient stated that at times she walked barefoot during her stay. The patient also reported that she already had a four-day antibiotic treatment course with amoxicillin 875 mg/Clavulanic acid 125 mg twice daily orally without any improvement of the symptoms.

**Figure 1:**
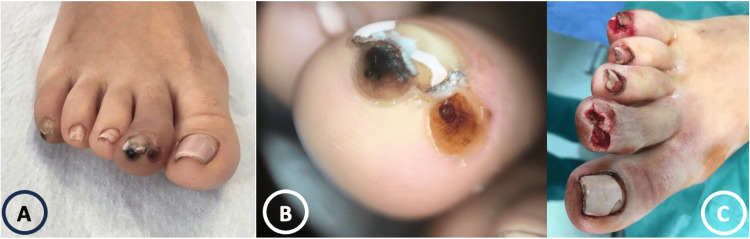
(A) Clinical, (B) dermoscopic, and (C) post-surgical images of the toe lesions

Dermatological examination revealed white to yellowish, slightly tender, firm papules, measuring 6-8 mm in diameter. The nodules had a crateriform dark brown central area and were surrounded by erythema and oedema (Figure 1A). No inguinal or other lymphadenopathy was present. The rest of the physical examination was unremarkable. The dermoscopic inspection of the lesions revealed a pigmented annular structure with a brown-to-black central pore, a white halo, and peripheral bluish-grey areas (Figure 1B). A surgical debridement of the lesion was performed (Figure 1C) and the extruded material was sent for microbiological and histological examination.

**Figure 2:**
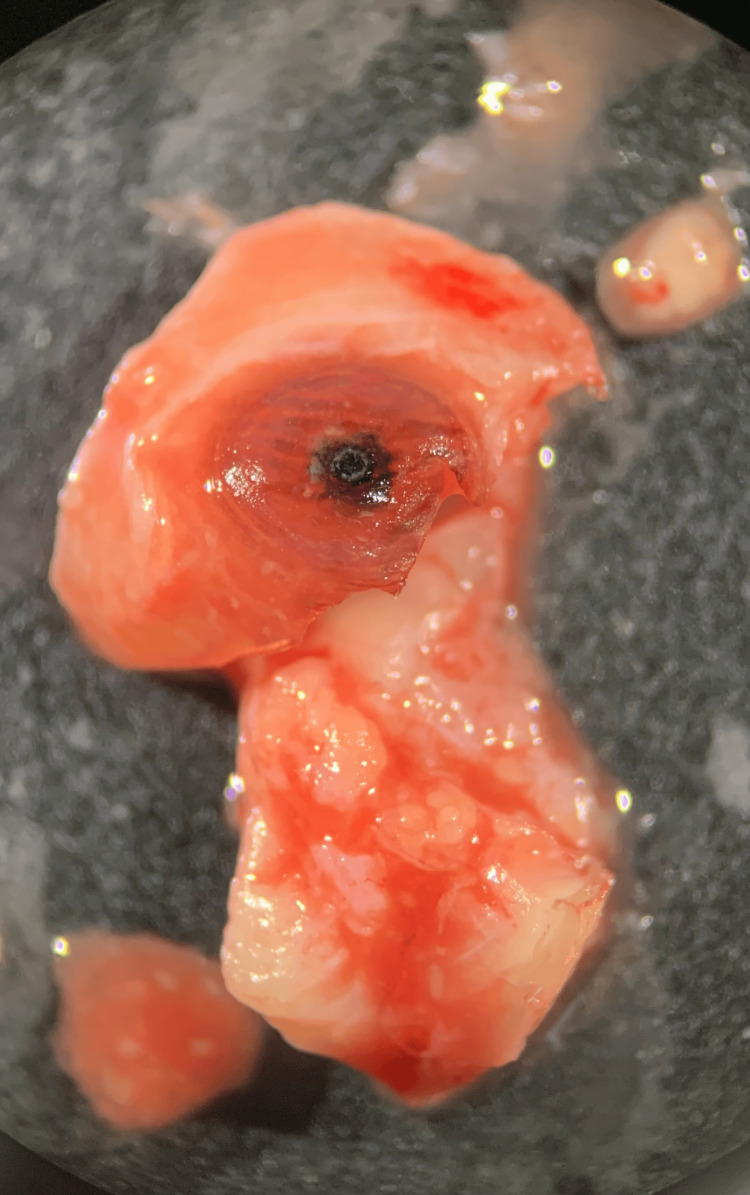
Host tissue surrounding the flea is thin, with a blackish spot where the flea’s posterior end was located

**Figure 3:**
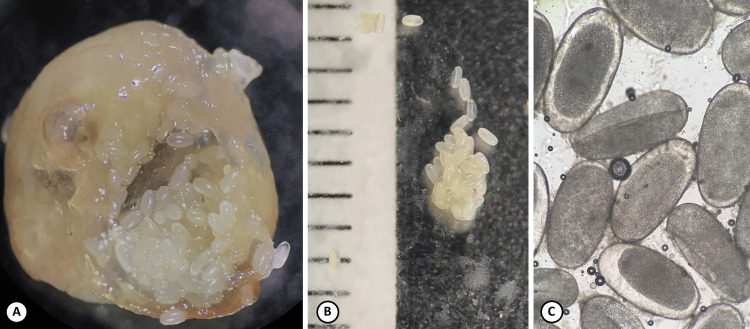
Tunga penetrans female abdomen containing numerous eggs A and B: images from a stereoscope (lines = 1mm). C: eggs under a light microscope, magnification 100x

**Figure 4:**
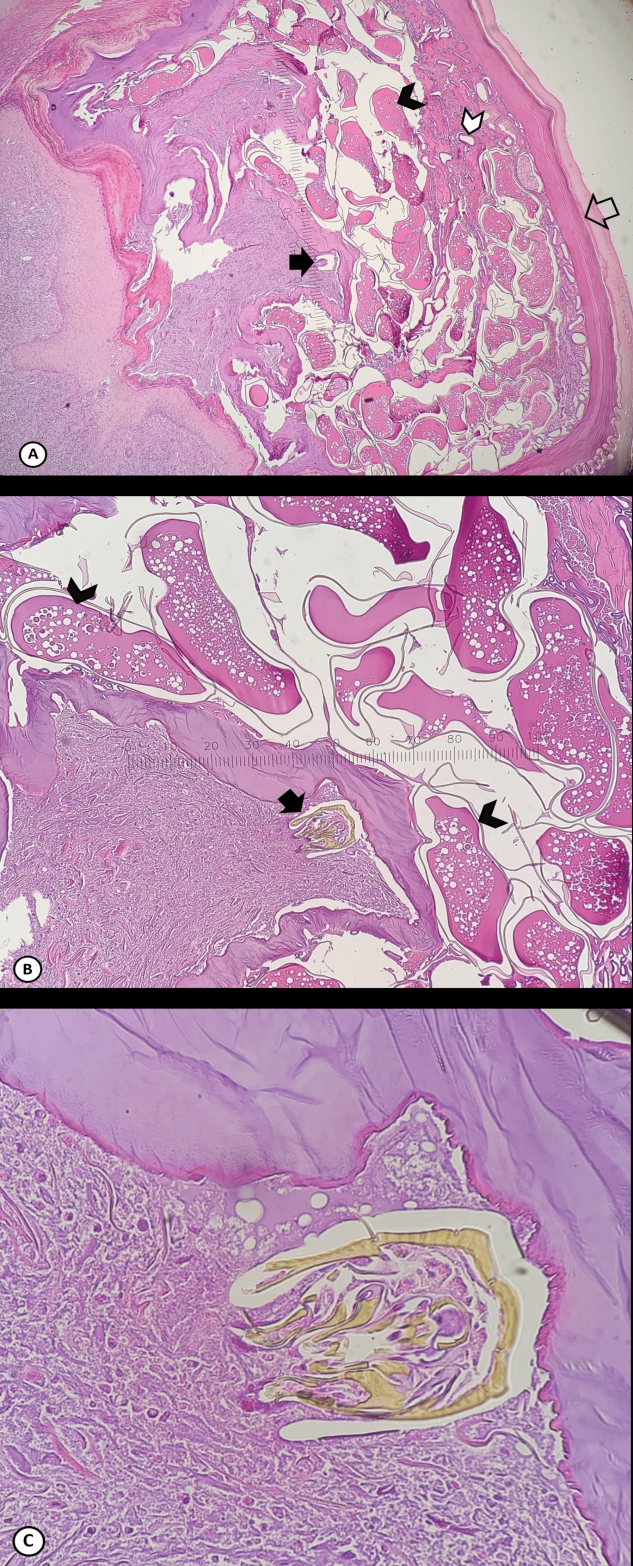
Tunga penetrans in the patient’s biopsy sample (hematoxylin and eosin) The arthropod parts are significantly deformed. A: Head (black arrow), eggs (black arrowhead), trachea (white arrowhead), exoskeleton (transparent arrow) (magnification 40x). The exoskeleton at the posterior part of the flea exhibits a pale-staining area between the inner and outer aspects, that has been previously described. B: Head (black arrow), eggs (black arrowhead) (magnification 100x). C: Flea head, exhibiting the mouthparts that are not stained with hematoxylin and eosin (magnification 400x).

Of the two nodules excised from the patient, one was examined under a stereoscope without processing, and the other was fixed, sectioned, and stained with hematoxylin and eosin. The nodule consisted of host tissue and the parasite. The embedded flea was approximately 0.8 cm in diameter and the skin around it was thin, with a blackish spot where the flea’s posterior end was located (Figure 2). The head was not visible. Identification was aided by observation of the extracted parasite under the stereoscope, specifically of the abdomen containing numerous eggs, each approximately 0.5 mm long (Figure 3), and the presence of the head, exoskeleton, tracheae, and eggs in the histologic sections (Figure 4).

Following the surgical procedure, the patient received a combined oral antibiotic treatment with ciprofloxacin 500 mg twice daily and clindamycin 300 mg thrice daily for 10 days. After completion of antibiotic treatment, the procedure of wound healing was satisfactory, and the patient was asymptomatic. The patient was followed up a month later, with a fully healed scar and without any symptoms or signs of infection recurrence. 

## Discussion

Aetiology

The genus *Tunga* (Phylum: Arthropoda, Class: Insecta, Order: Siphonaptera, Family: *Tungidae*) is divided into two subgenera: *Tunga* and *Brevidigita* [13-15]. Subgenus *Brevidigita* includes seven species, all infesting wild animals and rodents (*T. caecata*, *T. caecigena*, *T. callida*, *T. bossii*, *T. bonneti*, *T. libis*, *T. monositus*), while subgenus *Tunga* comprises six species (*T. penetrans*,* T. trimamillata*, *T. hexalobulata*, *T. travasossi*, *T. terasma*, *T. bondari*), of which only *T. penetrans* and *T. trimamillata* have been recognized as human and domestic animal ectoparasites [15,16].

Geographical distribution: epidemiology

*Tunga* spp. are mainly found throughout Latin America, but *T. penetrans* has also been introduced in sub-Saharan Africa [16]. *T. penetrans* has many names in different languages reflecting its geographical distribution, including “sand flea”, “jigger flea”, “chigger” or “chigoe” in English, “chique” in French, and “bicho do pè” in Portuguese; it is called “chica” in Venezuela and Columbia, “nigua” in Mexico, Bolivia and Ecuador, and “pique” in Argentina and Peru. The ectoparasite originated in South America, where it has been present since ancient times [10]. It was initially described in 1525 by the chronicler of the Indies, Gonzalo Fernández de Oviedo y Valdés from Haiti, as causing significant motility problems to invading European troops during the discovery of the Americas [10,15]. The sand flea was introduced to West Africa during the first half of the 18th century through trading routes, first in Senegal, then Gabon or Angola, and hence, to the whole of sub-Saharan Africa by the end of the 19th century. Although introduced to Asia by 1899 by workers or soldiers from the East African English colonies, the parasite has not established a presence there [10,15]. Nowadays, *T. penetrans* is endemic in Central and South America (from southern Mexico to northern Argentina, excluding Chile), the Caribbean islands, and sub-Saharan Africa [10,16]. *Tunga trimamillata* was first reported in Peru and Ecuador at the beginning of the 21st century, and most recently in Brazil, parasitizing several domestic animal species (cattle, goat, sheep, swine) and humans [10,17].

Tungiasis is related to poor sanitation and poor living conditions and is a typical poverty-associated disease. Prevalence rates in indigenous populations living in high-risk regions may reach 50% [18]. Tanzania, where the case subject acquired the infection, is highly endemic, and imported cases are frequently associated with visiting it. The infestation is sporadically reported in North America, Oceania, and Europe, mainly among immigrants and travellers returning from tropical areas. In Europe, the majority of cases are reported from Italy, nearly all of them in travellers returning from Latin America or sub-Saharan Africa. The current case is the second case of tungiasis reported in Greece, the first being reported by Dialynas et al. [19].

*T. penetrans* is one of the smallest fleas, with both sexes measuring approximately 1 mm long. It is characterized by a lack of genal and pronotal combs and can be distinguished from other fleas of medical importance by the very compressed anterior three (thoracic) segments and the scarcity of spines and bristles on the body [16,20].

*T. penetrans *eggs are released from lesions on animal paw pads or human feet on the floors of houses and in the soil; although they are found in various types of soil, dry and sandy soil is especially conducive to their development [16,19]. There are two instars, not three as occurs in other flea species. First-stage (L1) larvae hatch from the eggs, develop to L2, then L3 within 10-14 days, and consequently pupate. Pupae develop into adult fleas in about 5-14 days [21].

Adults penetrate the skin of their hosts through direct contact with sandy soil; thus, walking barefoot is the most common risk factor for infection. The flea can only jump up to 20 cm in height, which explains why the typical sites of infection are the toes, the periungual or subungual folds, and the plantar surface of the feet. Other sites like the fingers, breasts, and back are more commonly affected in inhabitants of endemic areas but rarely in travellers [4,20,22]. Both sexes are hematophagous, with the male penetrating the stratum corneum only for a few hours to feed and mate, whereas the female flea obligatorily burrows into the epidermis, where it becomes completely and permanently buried, with the exception of the tip of its abdomen, where the anus, genital opening, and large respiratory spiracles are located [15,16,20,23]. Fertilization can occur either in the environment or after penetration of the adult female flea into the host’s epidermis [15,16,20]. While the blood meal is being digested, the abdomen distends to an enormous size, reaching a final length of 1 cm from an initial length of less than 1 mm. This characteristic neosomy, a 10-fold hypertrophy of the abdomen, is due to constant egg production. After 8-10 days, the female starts releasing 150-200 eggs per day, and, after about five weeks, eventually dies. The life cycle from egg to adult usually lasts four to six weeks but may be as short as 18 days [4,15,21].

The typical lesion of tungiasis is caused by the female gravid flea entering the epidermis, and symptoms such as pruritus, pain, and foreign body sensation start to bother the host as the flea grows in dimensions. The area around the embedded flea becomes pruritic and inflamed, secondary infections may occur, and ulcerations with accumulation of pus may follow. The lesion typically presents as a yellow-white papule or nodule with a dark brown or black centre, where an opening on the skin can usually be observed, corresponding to the posterior part of the flea’s abdomen. The characteristic lesions are often accompanied by pain, pruritus, erythema, and oedema. After its death, the female remains embedded in the host, frequently inducing secondary bacterial or fungal infections, which, if ignored, may lead to deformation or loss of nails, foot disfigurement, ulcerations with extensive tissue necrosis, tetanus, and even gangrene. If left untreated, tungiasis may lead to difficulty in walking, autoamputation of digits, and complete immobility in severe cases. Differential diagnosis includes myiasis, arthropod bites, furuncles, abscesses, paronychia, viral warts, folliculitis, foreign body, early melanoma, and fungal granulomas. *T. penetrans* may be a nuisance but does not transmit any diseases.

The flea can be extracted using a sterile scalpel or needle by cutting the skin around the posterior end and pulling the entire flea out with tweezers. Fleas are best removed within the first few days of the infestation because after they become distended, they are hard to extract without becoming ruptured, increasing the risk of infection. Surgical extractions are not easy and sometimes local treatment with ivermectin or metrifonate (trichlorfon) lotions for in situ destruction of the flea is preferred [24]. Antibiotics may be required to treat concomitant bacterial infection. Prophylaxis of human tungiasis in endemic areas includes wearing closed shoes, using repellents like DEET (N,N-diethyl-meta-toluamide) on the skin, insecticidal treatment of the soil in infested houses, and the preventing animals from wandering inside and near houses.

In endemic areas, tungiasis is readily diagnosed by experienced clinicians during physical examination; therefore, no further diagnostic workup is necessary. In non-endemic areas, however, unfamiliarity with the parasite frequently results in a biopsy of the lesion. Biopsy specimens rarely exhibit textbook morphology, with an intact longitudinally aligned body and a head attached to it, but rather usually present an assembly of broken and deformed insect body parts, hindering definitive identification. Histopathologic differential diagnoses include ticks, mites, helminths, and fly larvae. Morphologic structures that may be observed in *T. penetrans* lesion biopsies include the head, exoskeleton, hypodermal layer, tracheae, digestive tract, striated muscle, posterior end, and eggs in various stages of development [12].

## Conclusions

As with other parasitic infections that are not endemic in developed countries, travel history and characteristic symptoms and findings can provide important clues for diagnosis. Specifically, a history of barefoot walking on sandy soil in tropical areas, and the presence of a nodule, approximately 6mm with a black center in the lower extremities should alert the clinician to the possibility of tungiasis. Microscopic and histological examination by an experienced microscopist will usually confirm the diagnosis.
